# Inspection of Piezoceramic Transducers Used for Structural Health Monitoring

**DOI:** 10.3390/ma10010071

**Published:** 2017-01-16

**Authors:** Inka Mueller, Claus-Peter Fritzen

**Affiliations:** Department of Mechanical Engineering, University of Siegen, Paul-Bonatz-Str., 9-11, 57076 Siegen, Germany; inka.mueller@uni-siegen.de or inka.mueller@aol.de

**Keywords:** piezoelectric transducers, piezoelectric wafer active sensors (PWAS), electro-mechanical impedance (EMI) spectrum, fault detection, diagnosis

## Abstract

The use of piezoelectric wafer active sensors (PWAS) for structural health monitoring (SHM) purposes is state of the art for acousto-ultrasonic-based methods. For system reliability, detailed information about the PWAS itself is necessary. This paper gives an overview on frequent PWAS faults and presents the effects of these faults on the wave propagation, used for active acousto-ultrasonics-based SHM. The analysis of the wave field is based on velocity measurements using a laser Doppler vibrometer (LDV). New and established methods of PWAS inspection are explained in detail, listing advantages and disadvantages. The electro-mechanical impedance spectrum as basis for these methods is discussed for different sensor faults. This way this contribution focuses on a detailed analysis of PWAS and the need of their inspection for an increased reliability of SHM systems.

## 1. Introduction

Aiming at a significant improvement of inspection quality, structural health monitoring (SHM) is an innovative approach of continuous or periodical and automated inspection and determination of the condition of a monitoring object. It can be used complementary to traditional non-destructive inspection (NDI) methodologies, leading to advantages for condition-based maintenance. The SHM system consists of the monitoring object itself with the transducers, the storage unit as well as the signal processing unit and the automated expert system for diagnosis (translated from [1]).

In [2] it is mentioned, that traditional condition monitoring systems, which are used for long term monitoring, need to have a self-check to ensure system reliability. For SHM systems this topic has long been neglected, assuming a perfectly working system in the first place. Especially for the industrial application this is not acceptable. Authorities request system checks to qualify systems and a high reliability is necessary to satisfy stakeholders. But also for the scientific work this system reliability is of major importance as Giurgiutiu mentions in ([3] p. 394) that “The integrity of the sensor and the consistency of the sensor/structure interface are essential elements that can make or break an experiment”.

Currently the attention on reliability of methods, used for SHM is increasing. Mentioning the keyword “probability of detection” (POD), methods for the detection of structural damages are evaluated. This is one necessary factor for describing the system reliability. Additionally a self-check of all included system components is essential comprising the inspection of the instrumented transducers.

Nevertheless, there has only been “little work on the sensor diagnosis, which is applicable to active sensing devices used in SHM applications” ([4] p. 76). Regarding sensor faults in general, a variety of methods to detect faulty sensors is based on the redundancy of sensor data. Using the data of redundant sensors, a model is built. Differences between the model and the measured data suggest sensor faults. These methods are called “hardware redundancy” [5]. Examples for these methods are given in [6], using null-space and a posteriori probabilities, as well as in [7,8] using principal component analysis (PCA). In [9] a decision tree-based on spatial and temporal correlation is used to achieve a classification of sensor faults in a wireless sensor system. Typical sensor faults, which are mentioned in [5,7] are bias, complete failure, drifting and precision degradation as well as gain, noise and constant values with noise. The mentioned methods are not system-related and not focused on applications of active acousto-ultrasonics-based SHM.

Other publications, which deal with SHM systems and use redundancy of the data to detect faulty sensors are [10,11]. The latter also shows how to replace the faulty signal by a modeled signal, based on the redundancy, using Auto-Regressive eXogenous (ARX)-models. In [12] the context of SHM is mentioned using multiple hypothesis tests for acceleration data. [13] also considers the effect of environmental and operational conditions when identifying faulty sensors with the mutual information concept. Common to all these approaches is the assumption that a structural damage leads to an effect in the signal of several sensors, while a defect sensor only changes the signal of the sensor itself. This assumption is not always valid as shown in [14]. Moreover all these approaches are purely mathematical without a relation to physical quantities. Alternatively it is possible to use “analytical redundancy” [5], using analytical or finite element models. As a high accuracy is necessary, the numerical costs for these methods are comparably high and small faults are difficult to detect.

All these methods are based on the normal measurements of the sensors and no additional measurements are necessary. This is an advantage as additional expenses are reduced. However, it also includes disadvantages as the distinction between structural damage and piezoelectric wafer active sensors (PWAS) fault is difficult especially for small defects and wide spread sensor networks. Moreover the typical sensor faults like drift or additional noise are not typical for defect PWAS. It is therefore necessary to take into consideration alternative concepts for PWAS inspection.

This work focuses on the piezoelectric transducers, which are used as sensors and actuators for active acousto-ultrasonics-based SHM systems. Single types of faults, like effects of the bonding layer, degradation and cracks, have been discussed in [15,16,17,18,19,20,21] using experimental data or modeling approaches. A detailed analysis for simple transducers with wrap-around electrodes is given in [22]. To identify these faults, in [23] a method based on the slope of the electro-mechanical impedance spectrum is shown. In [24] it has been shown that this method is very effective for some faults, while for other faults it proves to be inefficient as the damage is not detected. In [25] an alternative method is discussed, using the time reversal index, for the detection of sensor debonding and cracking. In [26,27] a method using multiple parameters extracted from the impedance spectrum, is used for the identification of faulty transducers.

Apart from this, the electro-mechanical impedance spectrum can also be used for the monitoring of properties of the structure, the PWAS is attached to. This is due to the fact that the electro-mechanical impedance of an attached PWAS is influenced by the mechanical and electrical properties of the PWAS and the bonding layer but also by the mechanical properties of the structure. If the structure should be monitored, only small frequency bands are used for the evaluation of the frequency spectrum. The resonances of the structure are focused in a frequency range apart from the resonances of the PWAS. For fatigue damage of the structure, this is shown in [14,28]. For truss or beam like structures, the use of the electro-mechanical impedance spectrum for structural damage detection is discussed e.g., in [29,30,31]. While in [30] the practical implementation for excavation support structures is focused, in [31] a modeling approach based on the spectral element method is shown. The practical implementation for multi-sensor measurement is detailed in [32]. The purpose of monitoring the structure using the electro-mechanical impedance spectrum has to be clearly separated from the purpose of identification of faulty transducers. In this paper different methods for PWAS inspection, based on the electro-mechanical impedance spectrum are shown and the context of PWAS faults including the effect on the wave field generated by the faulty PWAS is discussed in detail.

First of all, different frequently mentioned types of PWAS faults are discussed, including their effect on the generated wave field. Special focus is set on embedded transducers, which from the author’s point of view, are the future for industrial application as they are more robust to environmental influences. Secondly, the focus is put on the electro-mechanical impedance (EMI) spectrum, which is known to be sensitive to PWAS faults. A short overview on analytical models is given, supporting the sensitivity of this physical quantity. This is followed by an overview of methods for PWAS inspection, based on the EMI spectrum. Afterwards, the effects on the EMI spectrum are shown for the defects, discussed previously. Advantages and disadvantages are emphasized. This completes the comprehensive overview on possible faults of PWAS and summarizes as well as evaluates approaches for their inspection. On this basis it is possible to analyze PWAS in an SHM system and improve reliability issues, helping to mature SHM for industrial applications.

## 2. Basics of PWAS and PWAS Faults

The principle of operation of PWAS is based on the piezoelectric effect, which can be described with the following equations

(1) $$\begin{array}{cc} D_{i} & =d_{ikl}T_{kl}+\varepsilon_{ik}^{T}E_{k} \end{array}$$

(2) $$\begin{array}{cc} S_{ij} & =s_{ijkl}^{E}T_{kl}+d_{kij}E_{k} \end{array}$$

with stress *T*, strain *S*, elastic compliance *s*, dielectric constant ε , electric field *E*, dielectric displacement *D* defined as charge *Q* per unit area *A* at stress *T*, and piezoelectric constant *d*, which links the dielectric displacement *D* with stress *T* as well as the strain *S* with the electric field *E*, and is also called piezoelectric charge constant. The superscripts *T* and *E* represent a special state for the specific parameter, *T* represents zero stress, while *E* represents zero voltage, i.e. short circuit.

The piezoelectric effect couples mechanical and electrical effects, as an applied voltage leads to a mechanical strain and vice versa. While the first mentioned is used for actuating purposes, the latter coupling effect is used for sensing. A large variety of transducers exist. Different materials can be used, in this work Pb(Zr,Ti)O_3_ is used. Depending on the coercive field $E_{c}$ transducer materials can be separated into hard and soft lead zirconate titanate (PZT).

In this work two kinds of PWAS are investigated, discs with wrap-around electrode and embedded piezoelectric discs with encapsulation made from e.g., Kapton. The first type is called simple PWAS in this publication, while the other is called embedded PWAS.

### 2.1. Types of PWAS Faults

**Figure 1:**
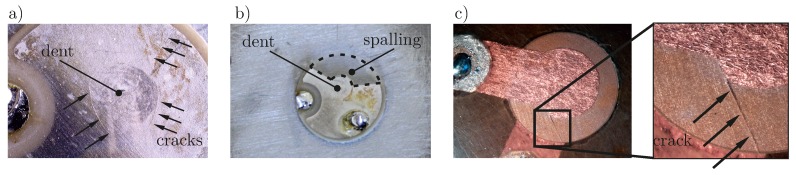
Types of Breakage: (**a**) Simple disc-shaped transducer (PIC 255, PI Ceramic GmbH, Lederhose, Germany) with cracks; (**b**) Simple disc-shaped transducer (PIC 255) with cracks and spalling, i.e., breakage into different pieces; (**c**) Embedded transducer (DuraAct, [34]) with crack.

A very common defect is the breakage of the piezoelectric transducer. It is the result of different causes, like an impact, caused by a tool drop, or high bending moments on the brittle ceramic material. Depending on the different types of PWAS the loading either results in cracks, breakage or spalling. While for simple transducers with wrap-around electrode spalling is common, this is hardly possible for embedded transducers, for which the embedding material holds together the broken parts. In [22] the fracture of transducers under cyclic loading is shown, in [17] cracks in the transducers, when impacts occur in the near vicinity on the structure are described. A PWAS breakage during a test campaign is documented in [33]. Gall et al. present the occurrence of cracks in encapsulated PWAS, caused by mechanical bending loads [16]. In Figure 1 three different types of breakage are depicted.

Another type of defect is the debonding of the transducer. It is the partial detachment of the transducer from the structure, which can be caused either due to bad bonding conditions or by a rupture or dissolving of the bonding layer. This defect type can be prevented with improved bonding conditions, like it is the case for co-bonding processes for transducers, which can be used to monitor structures made from carbon fiber reinforced thermoplastic (CFRP). The debonding is also mentioned in [35] as defect type. It is analyzed in [36], where the main goal is not the identification of faulty transducers, but the investigation of the effect of partially debonded transducers on the SHM algorithms.

Moreover a degradation of the material components, included in attached transducers is possible. Either the piezoelectric material or the bonding material can be focused. For secondary bonded transducers, which are glued with an adhesive component e.g., Z70 adhesive (HBM GmbH, Darmstadt, Germany), solvents like dimethylformamide can cause degradation. Moreover the exposure to high temperatures can cause degradation of the adhesive and the piezoelectric material. For transducers with capsulation also the degradation of the materials used for the encapsulation can degrade, leading to a changed behavior of the transducer. Degradation is mentioned in [22] as a damage caused by multiple cycles of mechanical loading and influence of environmental exposure.

A defect, which can be detected very easily, is the defect of the soldering connection. For industrial applications in most cases an integrated sensor system is preferred, which includes a reduced number of manual soldering points. Nevertheless this defect type has to be taken into account.

### 2.2. Effects of PWAS Faults on the Generated Wave

As PWAS are used to generate and sense waves for active acousto-ultrasonics-based SHM, the effect of PWAS faults on the generated wave field is important. The effect on the wave field is shown here for two exemplary damage types “breakage” and “debonding”. Using a laser Doppler vibrometer (LDV) it is possible to visualize the out-of-plane velocity field of the structure, which is excited by the PWAS motion. The details of the experimental setup to visualize the wave field are discussed in Section 6.

**Figure 2:**
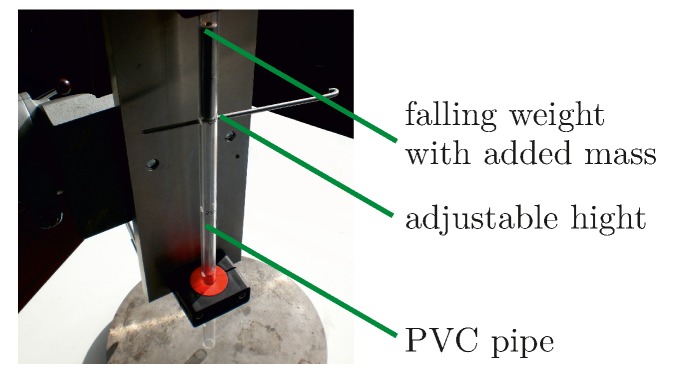
Tool drop setup.

To cause breakage caused by falling masses a small drop tower has been used. A mass of specific weight can be dropped on the sample from a specific height though a polyvinyl chloride (PVC) pipe, see Figure 2. This way the kinetic energy can be varied. The two transducers, PIC 255 (PI Ceramic GmbH, Lederhose, Germany), diameter 10 mm, thickness 0.5 mm, bonded to an aluminum plate, used for this impact test have been both impacted twice, each with a mass of 53.5 g from 350 mm height for the first impact and 450 mm height for the second impact. The first PWAS A shows cracks, the second PWAS B exhibits cracks and spalling. As input signal a windowed cosine train with five cycles and a central frequency of 85 kHz is to be used. For simple disc-shaped transducers, the location of the cracks is visible in the field of the maximum out-of-plane velocity measured on the surface below the attached transducer.

**Figure 3:**
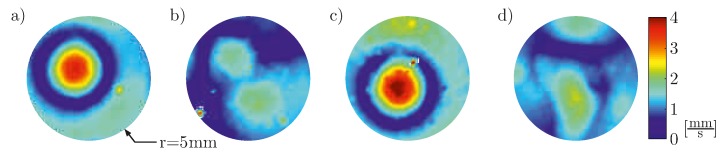
Maximum velocity field for simple disc-shaped transducers A and B (**a**) Baseline of A before impacting; (**b**) measurement after impacting A, leading to a transducer with cracks; (**c**) Baseline of B before impacting; (**d**) measurement after impacting B, leading to a transducer with cracks and spalling.

As it is shown in Figure 3 the impact has a similar effect for the generated wave field. It is not more pronounced for the case of spalling, but approximately the same. This leads to the assumption that the impact also effects the bonding layer between the PWAS and the structure leading to a reduced energy transfer also for the case of cracks only. Due to the wrap-around electrode and placing imprecision due to manual orientation of the plate, the maximum is not centered in Figure 3 also for the baseline measurements, repeatability of placement has been assured. Experiments with additional simple transducers without embedding exhibit similar results.

**Figure 4:**
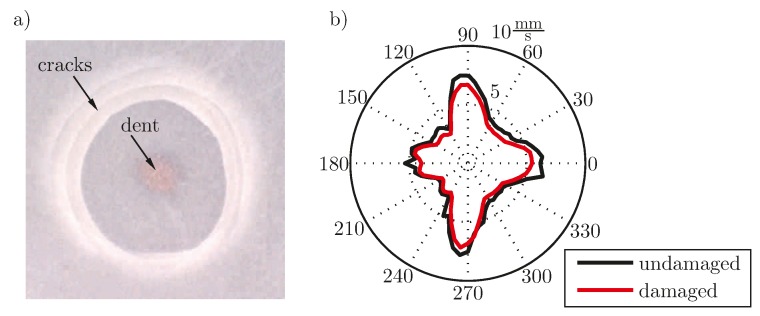
(**a**) Micrograph of the damage after grinding the upper surface of the piezoelectric wafer active sensors (PWAS); (**b**) Polar plot of the maximum out-of-plane velocity before and after the impact, measured at the structure.

**Figure 5:**
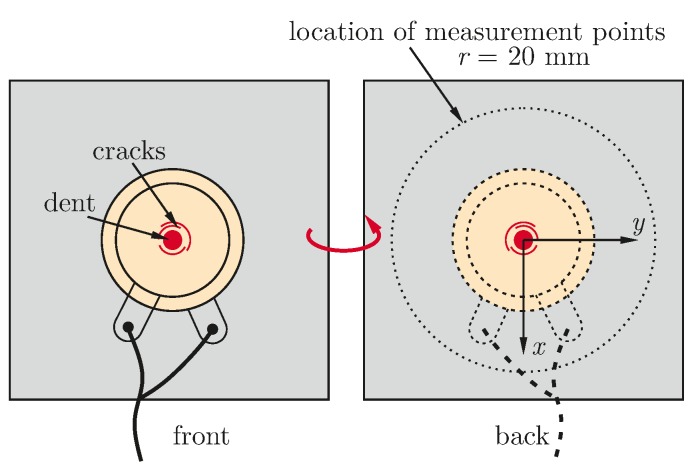
Location of the impact, leading to a dent, several crescent cracks and measurement locations to recognize changes of the velocity field in the structure.

The effect of breakage is less dominant for embedded transducers, as the encapsulation also transfers energy between different parts of a broken ceramic disc. Nevertheless an effect on the generated wave field can be recognized by measurement of the velocity field. For this experiment an embedded transducer, type DuraAct (PI Ceramic GmbH, Lederhose, Germany), see [34], has been impacted with a mass of 79 g droped from 570 mm height. A micrograph shows the damage pattern, in Figure 4a. For the introduced damage the differences of the generated wave field have been examined at a distance of 20 mm to the center of the PWAS. Figure 5 shows the location of the measurements, located on a circle with the center coinciding with the transducer’s center. A polar plot shows the differences of the velocity for this encapsulated transducer, bonded on a CFRP plate, before and after impacting the transducer from the top in Figure 4b.

The damage leads to a decreased maximum velocity while the main angular characteristics stay the same. Due to the highly anisotropic structural properties with [0/90] layup, the pristine state shows angular dependency with maxima oriented in the main directions of the layer. The results have been confirmed in a series of experiments with five additional impacted PWAS of the same type co-bonded on a CFRP panel. The influence of environmental effects such as temperature differences has been excluded. This has been done by measurement of a reference PWAS, which has not been damaged, showing that the signal of this transducer has not changed in the interval between the two measurements.

The effects of degradation of PWAS and adhesive material on the wave field are similar to the effects of breakage for simple disc-shaped transducers as well as for the embedded transducers. The wave field is less symmetric and the amplitude of the maximum velocity decreases. The similarity is also caused by the fact that a breakage often includes a partial degradation of the bonding layer.

**Figure 6:**
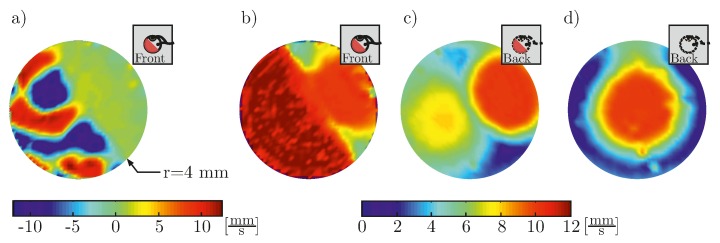
Velocity fields for a PWAS with 50% debonding: (**a**) At the top of the transducer, the velocity field at a given time step after the decay of the input signal shows the trapped energy; (**b**) While the maxima of the velocity field from the top show maximum values in the area of the debonding, where the PWAS can vibrate freely; in (**c**) it is shown, that this energy is not transferred into the structure; (**d**) For comparison, the maxima of the velocity field from the back for a fully bonded PWAS show almost symmetric pattern.

For the debonding of PWAS from the structure, additional effects on the generated wave field can be found. Results regarding these effects have been presented in [37]. In the experimental setup 12 simple PWAS (PIC 151, PI Ceramic GmbH, Lederhose, Germany, diameter 10 mm, thickness 0.5 mm) haven been bonded only partially on an aluminum plate exhibiting a debonding of 20% to 80% of the PWAS’s surface. For a simple disc-shaped transducer the debonded part stores energy, which is transferred to the structure only partly and delayed after the decay of the input signal. This effect can be visualized with the velocity field, measured at the top surface of the transducer and at the back of the structure beneath the transducer (Figure 6).

**Figure 7:**
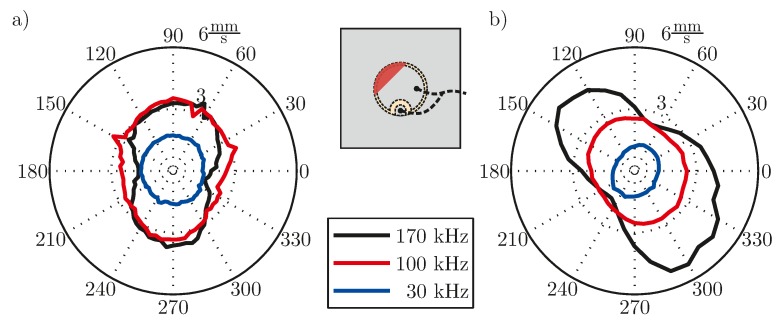
Polar plot of the maximum out-of-plane velocity for (**a**) a fully bonded transducer and (**b**) a 20% debonded transducer, measured at the structure on a circle around the PWAS center with a radius of 20 mm.

The effects, caused by debonding highly depend on the chosen actuation frequency and exhibit a strong angular dependency. Figure 7 shows the maxima of the velocities, measured at a circle around a PWAS for three different frequencies. The generated wave field for a simple disc-shaped transducer with wrap-around electrode is not symmetric for all frequencies in the case of a fully bonded transducer. As already mentioned in [38], this is caused by the wrap-around electrode which interrupts the axisymmetry. The shape of the maximum velocity polar plot changed due to the debonding. Depending on the frequency and the angle, the maximum velocity can even be higher than for the case of the fully bonded transducer.

It can be concluded that the different PWAS faults have a significant effect on the wave propagation. The changed wave propagation will lead to changes in the result of an SHM system, based on active acousto-ultrasonics, which use the PWAS for actuation and sensing purposes. Depending on the method used for acousto-ultrasonics-based SHM, these effects will have an impact on the statement about the health of the structure and might lead to false alarms, as not the structure but the PWAS is defective. It is therefore highly recommended to check the transducers to ensure the reliability of the SHM system.

## 3. The Electro-Mechanical Impedance (EMI) Spectrum of PWAS and Its Modeling

It has been shown e.g., in [15,23,24] that the EMI spectrum and especially the susceptance, which is the imaginary part of the admittance, as the reciprocal of the impedance, is sensitive to PWAS damages. Electrical and mechanical parameters influence this quantity, leading to the fact that a change of these parameters due to PWAS faults also changes the spectrum. Before showing the impact of PWAS faults on the EMI spectrum in the next section, this section gives an overview of how to model the EMI, unveiling the influence of different parameters on the EMI. This enables to understand that it is damage sensitive. Nevertheless, modeling includes making assumptions, e.g., axisymmetry, which makes it difficult to model the EMI spectrum of damaged PWAS.

The most simple way to model the impedance by neglecting any mechanical deformations is by modeling the PWAS as a capacitor, leading to

(3) Y(ω)=iωC

with

(4) $$C=\varepsilon_{33}\frac{A}{h}$$

and *A* being the area of the PWAS, perpendicular to the poling direction, *h* being the thickness in poling direction, taken as 3 as well as $\varepsilon_{33}$ being the dielectric constant in poling direction.

The impedance spectrum for a free circular transducer, not attached to a structure, taking into account mechanical deformations can be modeled with

(5) $$Y\left(\omega \right)=i\omega C\left(1-k_{p}^{2}\left(1-\frac{\left(1+\nu \right)J_{1}\left(k\cdot r_{\mathrm{PWAS}}\right)}{k\cdot r_{\mathrm{PWAS}}J_{0}\left(k\cdot r_{\mathrm{PWAS}}\right)-\left(1-\nu \right)J_{1}\left(k\cdot r_{\mathrm{PWAS}}\right)}\right)\right)$$

Bessel functions of the first kind $J_{n}$ are necessary to calculate the admittance. A detailed derivation can be found in [3]. The resonances are discussed also in [39]. Here $k=\frac{\omega }{c}$ is the wave number, *c* is the wave speed, $k_{p}$ is the planar coupling coefficient, $r_{\mathrm{PWAS}}$ is the radius of the PWAS disc and ν is the Poisson’s ratio of the piezoelectric material.

A model, based on similar assumptions also exists for strip like transducers [39]. A model for a bonded transducer, which includes the impedance of the structure $Z_{s}$ and of the PWAS $Z_{p}$ is given in [40]. For small wave number *k* it simplifies to

(6) $$\begin{array}{cc} Y\left(\omega \right) & =i\omega \frac{wl}{h}\left(\varepsilon_{33}^{T}-\frac{Z_{s}}{Z_{s}+Z_{p}}d_{31}^{2}c_{22}^{E}\right) \end{array}$$

with *w*, *l* and *h* being the PWAS width, length and thickness respectively and $c_{22}$ being the selected component from the matrix of elastic moduli. This model relies on the simplification that the PWAS can be modeled as a passive material, which has no electric coupling. It is suitable for applications putting the focus on the influence of structural changes on the impedance in the frequency range lower than the first PWAS eigenfrequency.

In [3] a sophisticated model for the susceptance of an attached circular PWAS, including the electric coupling, is given:

(7) $$\begin{array}{cc} Y & =i\omega C\left(1-k_{p}^{2}\left(1-\frac{\left(1+\nu \right)J_{1}\left(\varphi_{a}\right)}{\varphi_{a}J_{0}\left(\varphi_{a}\right)-\left(1-\nu \right)J_{1}\left(\varphi_{a}\right)+\chi \left(\omega \right)\left(1+\nu \right)J_{1}\left(\varphi_{a}\right)}\right)\right) \end{array}$$

with

(8)C=ε33πrPWAS2h(9)φa=ωcrPWAS(10)kp2=2d312ε33s11E1−ν(11)χ(ω)=kstr&adhkPWAS(12)kPWAS=tarPWASs11E1−ν

For the calculation of the admittance the capacitance can be calculated using the radius of the PWAS $r_{\mathrm{PWAS}}$ and its thickness *h*, the coupling coefficient $k_{p}$ with compliance $s_{11}^{E}$ as well as the quotient χ of the structure and adhesive and PWAS’s stiffness. $\varphi_{a}$ is a variable combining ω , *c* and $r_{\mathrm{PWAS}}$ .

Giurgiutiu gives a detailed derivation of χ for a disc-shaped structure, including a frequency dependency for $k_{\mathrm{str}}$ but neglecting $k_{\mathrm{adh}}$ . This is necessary for using the EMI for structural damage detection. As here the PWAS itself is focused, $k_{\mathrm{str}}$ shall be modeled as general as possible. Instead a more detailed modeling of the bonding layer is necessary.

In [41] a detailed derivation is given, leading to $k_{\mathrm{adh}}$ . With this result, the influence of the bonding line can be included in $k_{\text{str\&adh}}$ , which is inserted to find χ for the modeling of the susceptance *B* as imaginary part of the admittance *Y* in Equation (7).

(13) $$\begin{array}{c} \;k_{\mathrm{adh}}=\frac{G_{\mathrm{adh}}}{2}\frac{{\left(\frac{\omega }{c\sqrt{\alpha }}\right)}^{2}}{\sinh^{2}\left(\frac{\frac{\omega }{c}h_{\mathrm{adh}}}{\sqrt{\alpha }}\right)}\left(\frac{\sinh\left(\frac{\frac{\omega }{c}h_{\mathrm{adh}}}{\sqrt{\alpha }}\right)}{2\frac{\omega }{c\sqrt{\alpha }}}+h_{\mathrm{adh}}\right)\\ \;\;\cdot \frac{1}{2}\frac{\frac{\omega }{c}r_{\mathrm{PWAS}}J_{0}^{2}{\left(\frac{\omega }{c}r_{\mathrm{PWAS}}\right)}^{2}-2J_{0}\left(\frac{\omega }{c}r_{\mathrm{PWAS}}\right)J_{1}\left(\frac{\omega }{c}r_{\mathrm{PWAS}}\right)+\frac{\omega }{c}r_{\mathrm{PWAS}}J_{1}^{2}\left(\frac{\omega }{c}r_{\mathrm{PWAS}}\right)}{\frac{\omega }{c}J_{1}^{2}\left(\frac{\omega }{c}r_{\mathrm{PWAS}}\right)} \end{array}$$

Within this equation $G_{\mathrm{adh}}$ is the shear modulus of the adhesive and $h_{\mathrm{adh}}$ is the thickness of the adhesive layer. α is defined as $\frac{1}{2}\left(1-\nu_{\mathrm{adh}}\right)$ using Poisson’s ratio of the adhesive material $\nu_{\mathrm{adh}}$ .

Using this model, it is possible to analyze the effect of different parameters like the thickness of the adhesive layer or the piezoelectric coefficient $d_{31}$ . It helps to understand the relation between characteristic features of the spectrum and parameters of the experimental setup. Nevertheless the modeling of faulty transducers is limited due to assumptions like axisymmetry.

## 4. Effects of PWAS Faults on the EMI Spectrum

As it has been indicated, PWAS faults have an effect on the susceptance spectrum. This effect does not only depend on the type of fault but also on the type of transducer. It has been shown in Section 2.2 that if a PWAS is impacted e.g., by a falling object, this leads to different consequences for the generated wave field for the case of an embedded or a simple PWAS.

Cracks have been introduced for two simple PWAS and one embedded PWAS. In the experimental investigation, the drop tower as described in Section 2.2 was used for the simple PWAS. The crack was introduced into the embedded PWAS, co-bonded on a CFRP strip via bending. All treatments lead to cracks in the piezoelectric material. For the faults introduced, susceptance spectra are depicted to show the different effects on the spectra.

**Figure 8:**
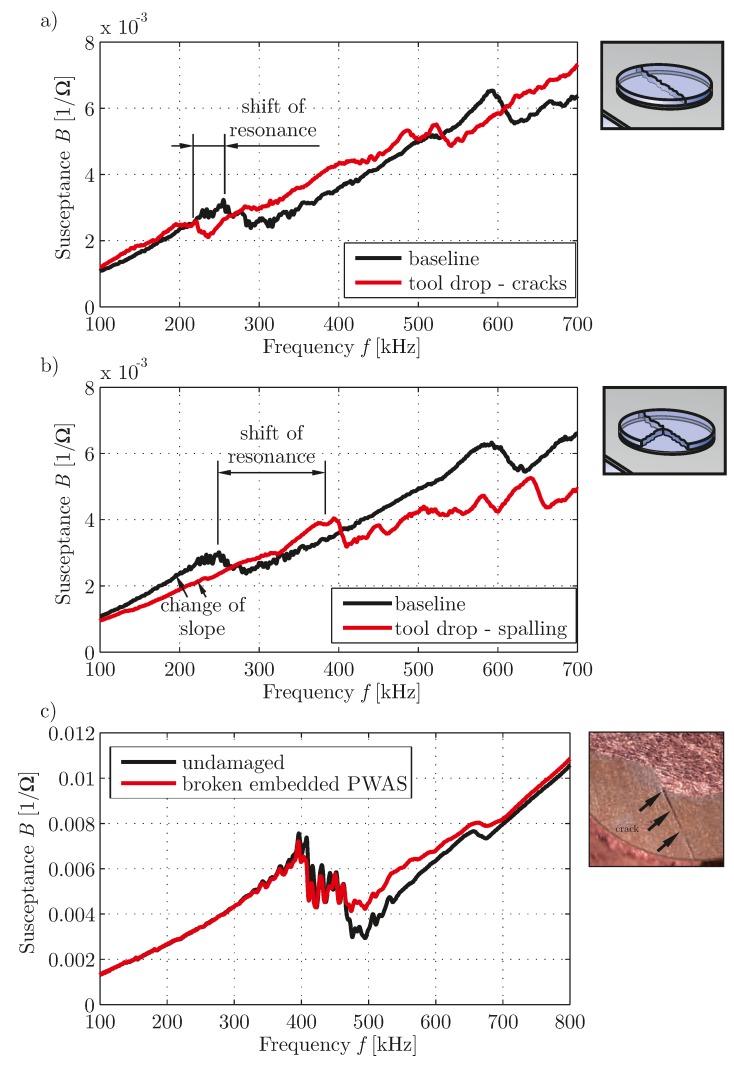
Susceptance spectra for the fault breakage: (**a**) Simple disc-shaped transducer, PIC 255, diameter 10 mm, thickness 0.5 mm, bonded to an aluminum plate, with cracks; (**b**) simple disc-shaped transducer, PIC 255, diameter 10 mm, thickness 0.5 mm, bonded to an aluminum plate, with cracks and spalling; (**c**) embedded transducer, PIC 255 material, type DuraAct, see [34], with cracks.

As shown in Figure 8, breakage leads to a change of the spectrum in the frequency range of resonance for all cases. While for a simple disc-shaped transducer a shift of the resonance is caused by cracks and a shift of the susceptance slope is caused by spalling, for the embedded transducer only the behavior in the frequency range higher than the PWAS resonance is changed.

Using the models, described in Section 3, the effects of damage on the EMI spectrum shall be discussed. The crack without spalling of the simple PWAS changes the stiffness of the PWAS, which is part of χ(ω) , but also of $k_{p}$ , as here the compliance is different at the location of the crack. This leads to a change of resonance frequency. The slope is not changed, as it is mainly influenced by the capacitance *C*, whose influencing parameters are not changed by the cracks without spalling. For the case of spalling, the active area of the PWAS is changed, which effects the capacitance. This leads to a decrease of the susceptance slope. The embedding material of the embedded PWAS ensures a prepressure also in the case of cracks. The change of the stiffness is therefore much smaller than for the simple PWAS with cracks. Nevertheless, an influence on the susceptance spectrum is visible. Cracks therefore have an influence on the EMI spectrum.

To simulate a degradation of the embedding material as well as the adhesive layer, dimethylformamide has been used. For the treatment a silicone ring was applied around the transducer and a Teflon sheet was placed on the wet silicone to build a barrier for the volatile substance. After drying the chemical liquid has been injected penetrating the sheet. The injection was repeated every two or three days, as soon as the liquid volatilized.

**Figure 9:**
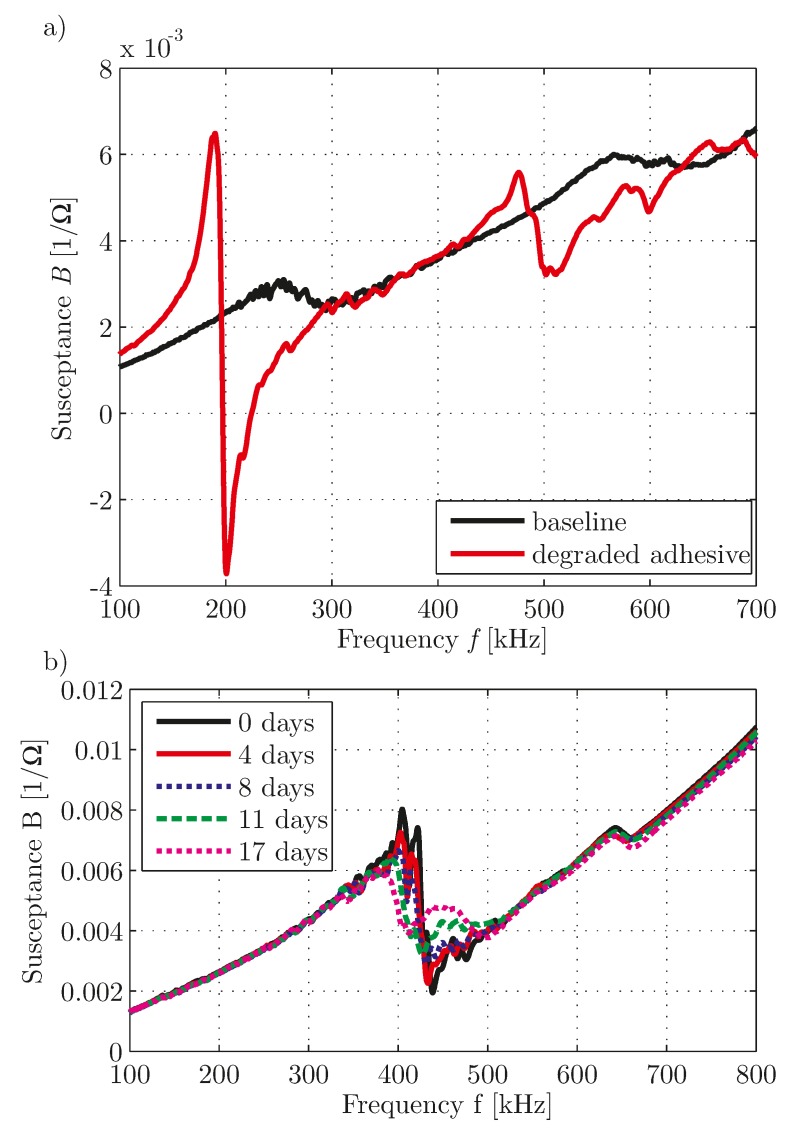
Susceptance spectra for PWAS with degradation caused by treatment with dimethylformamide: (**a**) Simple disc-shaped transducer, PIC 255, diameter 10 mm, thickness 0.5 mm, bonded to an aluminum plate; (**b**) embedded transducer, material of PIC 255, type DuraAct, see [34], cobonded to a CFRP strip, duration of treatment in days is given as a legend

In Figure 9, the spectra of a simple and a co-bonded embedded PWAS treated with dimethylformamide to create degradation are depicted. The effect for the simple disc-shaped transducer in Figure 9a is very similar to the effect, caused by a contaminated bonding surface. The resonance is much more pronounced and shifted to lower frequencies, because the vibration of the PWAS is more similar to the free vibration. This effect has also been shown by [15]. The effect for the embedded transducer differs. Here the effect of the resonance is less pronounced. From this it can be assumed that mainly the embedding has been degraded due to the treatment with dimethylformamide while the bonding is still intact. For both damage cases the general linear trend of the spectrum is not changed.

Based on this knowledge, methods for PWAS inspection based on the electro-mechanical impedance spectrum are discussed.

## 5. Methods for PWAS Inspection Based on the EMI Spectrum

The most common method for PWAS inspection based on the EMI spectrum is to monitor the susceptance slope. The calculation of a slope coefficient in a frequency range much below the range, which is influenced by the resonance, is detailed in [23]. This method has proven to be effective for simple transducers and large breakages with spalling. In [24] it has been shown that especially for embedded transducers, this method is not very sensitive. As long as the capacitance did not change due to a full detachment of a part of the transducer, a change of the slope coefficient is hardly detectable. Moreover the method is highly sensitive to temperature changes, which makes a temperature compensation necessary. The method is very easy to use and comfortable, if large breakages have to be detected.

Based on this concept in [26] an extended method has been described. Additional to the susceptance slope coefficient other parameters like the resonance frequency etc. are extracted from the susceptance spectrum. For this method a larger frequency spectrum has to be measured. Due to the inclusion of more information, it is possible to detect those damages, for which the slope does not change. For the evaluation of an inspection result a principal component analysis (PCA) of baseline data is done, leading to a damage index. A difficulty of this method is the reliable extraction of the parameters from the susceptance spectrum even for the damaged case, as the frequency range, used for searching the resonance, might change due to the damage.

Based on the analytical model, it is possible to evaluate the state of PWAS with a model-based method, which is described in [41]. To use this method, the model is adapted to experimental data via fitting of parameters. The fitted parameters are used for the analysis of the state of the transducer. While this method is advanced and includes a detailed analysis of the transducer, this is also the disadvantage, as a lot of knowledge about the transducer is required and the model can represent damages, which are in most cases not axisymmetric, only limited.

Using the susceptance spectrum, an easy but effective method is based on the calculation of the correlation coefficient. It can be calculated using the covariance matrix *V*, giving the covariance of the two spectra, which should be compared, the baseline $B_{0}\left(f\right)$ and the tested one $B_{t}\left(f\right)$ . *V* therefore is a 2 × 2 matrix. Before calculating the covariance matrix, the susceptance spectrum is smoothed. This smoothness helps to minimize the effect of structural changes. For this application, the correlation coefficient CC varies between 0 and 1 with 1 showing best matching. From this parameter a damage index $DI_{CC}$ can be calculated easily by its subtraction from 1.

(14)CC=V12V11V22(15)DICC=1−|CC|

In general the correlation coefficient might vary between −1 and 1. As the general slope is always positive, the range between −1 and 0 can be neglected. The damage index $DI_{CC}$ will therefore vary between 0 and 1 even without taking the absolute value. Resulting from mathematics the correlation coefficient is 1 for two susceptance curves, which only differ by a proportional change of the spectrum ( $B_{t}=\alpha B_{0}$ with α being a real constant). Therefore, $DI_{CC}$ cannot indicate a changed slope. With $DI_{CC}$ the focus is on those parts of the spectrum, which do not exhibit a constant slope. The resonance and its effects on the susceptance spectrum vary differently for different types of PWAS defects. This changes are used and recognized, when using $DI_{CC}$ . For temperature compensation it is favorable that the change of the slope is not detected by $DI_{CC}$ , as the main effect of a temperature change is a change of the slope. But a temperature change also leads to a shift of the resonance frequencies. This shift causes an increase of $DI_{CC}$ . It is therefore necessary to have a baseline for several temperatures in the temperature range under consideration. In [24] this method has proven to be very effective for the detection of minor and major PWAS faults. A final short example taken from this context shows the advantages of $DI_{CC}$ for embedded transducers with cracks, introduced by bending.

**Figure 10:**
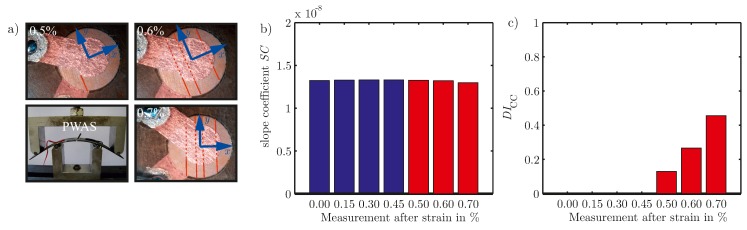
(**a**) Experimental setup and micrographs of the damage states of an embedded PWAS, type DuraAct, see [34], cracks caused by bending in a four-point bending test; (**b**) Calculated slope coefficient SC for measurements at different damage states; (**c**) Calculated damage indicator $DI_{CC}$ for measurements at different damage states. The bars are marked blue for the undamaged and red for the damaged state.

In Figure 10a the experimental setup for a four-point bending test is shown, as well as the cracks, caused by the high bending loads with a strain of up to 0.7%. The first crack is visible at 0.5% strain. As can be seen in Figure 10b,c, while the slope coefficient SC only shows very minor changes, the damage indicator $DI_{CC}$ for PWAS inspection clearly separates the measurements of the undamaged and the cracked PWAS.

## 6. Materials and Experimental Setup

For the experimental results shown in this paper, different methods have been used, which are detailed here. To give an impression on the effects of defect PWAS on the generated wave field, the out-of-plane velocity has been measured with a laser Doppler vibrometer (LDV). For the different experiments different vibrometers have been used.

The 1D helium-neon LDV used in this work is a measurement device from Polytec from 1998. The device is composed of a compact laser vibrometer (CLV) 700 measurement head with a CLV 800 laser unit, connected to a vibrometer controller CLV 1000. This controller is equipped with an output module CLV M002, a decoder module CLV M030 and an input module CLV M200. The equipment is based on analog technique and gives an output of ±10 V proportional to the measured velocity in direction of the laser beam.

Alternatively a digital short wave infrared (SWIR) 1D LDV from Optomet was used. This device consists of the Nova Basis with a dual fiber system. It includes a velocity decoder D-VD-1N. A passive fiber optic with a short range objective lens (OBJ-DF-SR) was used as dual fiber head. The system gives an output of ±2 V proportional to the measured velocity in direction of the laser beam.

Both 1D laser can be mounted on a *x*-*y* table with a separate controller, controlling the stepping motors of the two axes (stepping motor controller C116-4 from isel). It is controlled via a serial connection using MATLAB (Version 7, MathWorks Inc., Natick, MA, USA). The *x*-*y* table includes a vertical clamping of the measurement samples at the top of the measurement range, which enables a clamping at one edge of the sample.

**Figure 11:**
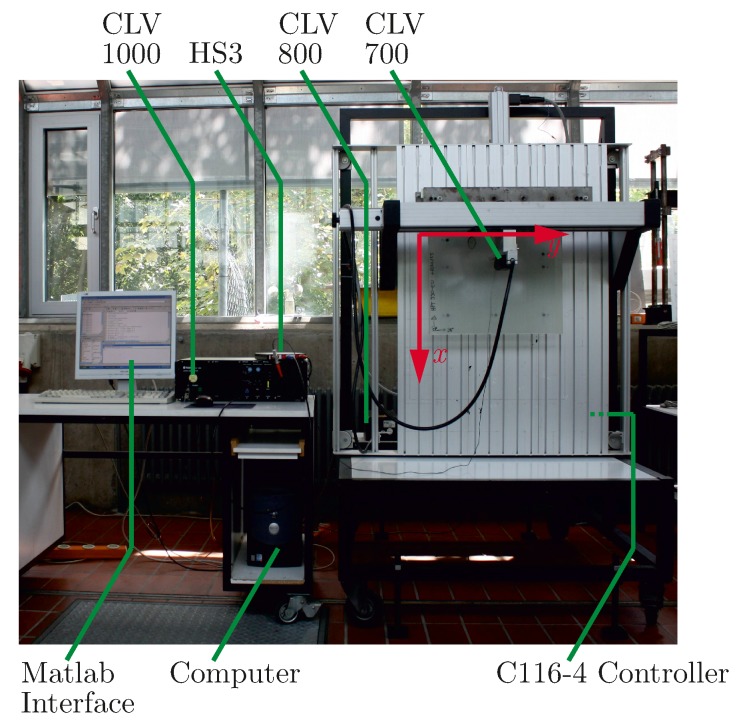
Experimental setup of the 1D LDV mounted on an *x*-*y* table.

The data acquisition is also controlled with MATLAB. A portable two channel measurement device with arbitrary waveform generator, called Handyscope HS3 from TiePie engineering (Sneek, The Netherlands) builds the connection between vibrometer controller and computer with MATLAB. Moreover it is used to actuate the PWAS, which generates the wave and its velocity field. This way also the triggering is controlled. The experimental setup is displayed in Figure 11. With this equipment the out-of-plane velocity can be measured pointwise within a defined area.

For the measurement of the EMI spectrum the PZT Inspector, a measurement device, which has been developed at the University of Siegen in the working group, led by the second author, is used. It enables a low cost automatic measurement of up to 32 channels. A detailed description of the device is given in [42]. For the measurement of the EMI the electrical measurement circuit, which is presented in [43] is applied in the device. A frequency sweep is used as input signal. The measured output is proportional to the current of the PWAS. As the device is connected to the PWAS via cabling, the measured EMI spectrum includes the influences of the connecting cables.

## 7. Conclusions

The need for PWAS inspection in active acousto-ultrasonic-based SHM systems is justified, as is has been shown, that PWAS faults influence the generated wave field, which leads to a changed performance of the SHM system for structural damage detection. Depending on the type of transducer as well as type and severity of PWAS fault, the effects on the generated wave field vary. For the same fault and transducer additional variation with the angle has been documented. This also substantiates that it is important to take into consideration the effects, PWAS faults have on the SHM system to identify its severity. Defect sizes for the PWAS like crack length or relative debonded area are of minor importance. Only a PWAS fault which effects the SHM system, needs to be detected. For the detection of PWAS faults, the suscpetance as imaginary part of the admittance has proven to be sensitive. The effect of different faults on the spectrum has been shown. A short overview on physical modeling of the EMI shows, why faults have an impact on this quantity, which includes electrical and mechanical parameters. Different methods for PWAS inspection, based on the EMI have been introduced, listing advantages and disadvantages as well as necessary references to case studies. For the performance assessment it will be necessary to combine the output of the PWAS inspection method with the effects, PWAS faults have on an SHM system. An approach for this combination is given in [44]. It is based on the concept of probability of detection. Future research activities shall include the combination of a designed SHM system with a defined PWAS inspection method to analyze the reliability of the SHM system with its different aspects in detail. 

## Abbreviations

The following abbreviations are used in this manuscript:

EMI	Electro-Mechanical Impedance Spectrum
LDV	Laser Doppler Vibrometer
NDI	Non-Destructive Inspection
PCA	Principal Component Analysis
POD	Probability of Detection
PWAS	Piezoelectric Wafer Active Sensor
SHM	Structural Health Monitoring
SWIR	Short Wave InfraRed
